# Impulse Control Disorders and Effort‐Based Decision‐Making in Parkinson's Disease Patients with Subthalamic Nucleus Deep Brain Stimulation

**DOI:** 10.1002/mdc3.14318

**Published:** 2025-01-03

**Authors:** Deborah Amstutz, Katrin Petermann, Mario Sousa, Ines Debove, Marie Elise Maradan‐Gachet, Lena C. Bruhin, Andreia D. Magalhães, Gerd Tinkhauser, Andreas Diamantaras, Julia Waskönig, Lenard Martin Lachenmayer, Claudio Pollo, Dario Cazzoli, Tobias Nef, Masud Husain, Paul Krack

**Affiliations:** ^1^ Department of Neurology Inselspital, University Hospital Bern, University of Bern Bern Switzerland; ^2^ Graduate School for Health Sciences, University of Bern Bern Switzerland; ^3^ ARTORG Center for Biomedical Engineering Research, University of Bern Bern Switzerland; ^4^ Department of Neurosurgery Inselspital, University Hospital Bern, University of Bern Bern Switzerland; ^5^ Nuffield Department of Clinical Neurosciences Oxford University Oxford United Kingdom

**Keywords:** Parkinson's disease, deep brain stimulation, impulse control disorders, effort‐based decision‐making, neuropsychiatric symptoms

## Abstract

**Background:**

Impulse control disorders (ICD) are common side effects of dopaminergic treatment in Parkinson's disease (PD). Whereas some studies show a reduction in ICD after subthalamic nucleus deep brain stimulation (STN‐DBS), others report worsening of ICD or impulsivity.

**Objective:**

The aim was to study ICD in the context of STN‐DBS using an objective measure of decision‐making.

**Methods:**

Ten PD patients performed an effort‐based decision‐making task alongside neuropsychiatric and cognitive evaluation before and 4 months after STN‐DBS. Further, 33 PD patients underwent the same experimental procedures just once after an average 40 months of chronic STN‐DBS. Participants were examined preoperatively in the medication *on* state and postoperatively in the medication *on*/stimulation ON state. Mixed linear models were used to assess the impact of ICD and STN‐DBS on acceptance rate and decision time in the task while controlling for motor symptom burden, cognitive measures, and dopaminergic medication.

**Results:**

Results revealed an increased willingness to exert high levels of effort in return for reward in patients with ICD, but acceptance rate was not modulated by chronic STN‐DBS. Further, ICD, cognitive processing speed, and STN‐DBS were all identified as positive predictors for faster decision speed. ICD scores showed a tendency to improve 4 months after STN‐DBS, without an increase in apathy scores.

**Conclusions:**

Chronic STN‐DBS and ICD facilitate effort‐based decision‐making by speeding up judgment. Furthermore, ICD enhances the willingness to exert high levels of effort for reward. Both STN‐DBS and dopaminergic medication impact motivated behavior and should be titrated carefully to balance neuropsychiatric symptoms.

Impulse control disorders (ICD) have a high prevalence in Parkinson's disease (PD)^1^, ^2^ and are characterized by a repetitive execution of pleasurable behaviors despite negative consequences.^3^ They include pathological gambling, compulsive shopping, hypersexuality, and binge eating, as well as related behavioral disorders such as excessive hobbyism, punding, and overuse of dopaminergic medication.^4^, ^5^ Subthalamic nucleus deep brain stimulation (STN‐DBS) is a safe and effective treatment for advanced PD, alleviating motor symptoms, enabling a decrease in the use of dopaminergic medication, and improving quality of life.^6^

The effect of STN‐DBS on ICD remains debated, with some studies showing a reduction in ICD associated with tapering of dopaminergic medication^7^, ^8^, ^9^ and others reporting no change^10^ or worsening of ICD in some patients.^11^, ^12^ Importantly, improvement in ICD may come at a cost, because some meta‐analyses show a postoperative increase in apathy.^13^, ^14^ On the contrary, studies investigating impulsivity ON and OFF stimulation revealed that STN‐DBS itself can improve behavioral flexibility while preserving negative feedback learning and delay discounting.^15^ However, in tasks involving competing choices, STN‐DBS leads to more impulsive decision‐making.^16^, ^17^ Studies on motor inhibitory control have led to conflicting results.^18^, ^19^, ^20^, ^21^, ^22^ Notably, motor disinhibition has been associated mainly with ventral stimulation of the STN, whereas dorsal stimulation of the STN can improve inhibitory control.^21^, ^22^, ^23^, ^24^, ^25^ Regarding mood, STN‐DBS has been shown to have acute positive effects even in the absence of dopaminergic medication.^26^

Research on ICD in PD is challenging, as symptoms are influenced by different factors, including postoperative management of stimulation parameters and medication dose,^27^ the severity of mesolimbic denervation,^28^ and the exact localization of electrodes within the STN.^25^ Conventional clinical impulsivity measures may not be useful markers of ICD, but differences in reward processing can be predictive of ICD.^29^ Thus far, clinicians have to largely rely on self‐report scales to assess ICD, which can be inaccurate due to some patients denying or lacking awareness of those behaviors.^30^

Effort‐based decision‐making tasks have been developed to quantify motivated behavior in the healthy population and a variety of psychiatric illnesses.^31^ The roots of effort‐based decision‐making lie in decision theory, which assumes that motivated behavior is a function of both the reward to be obtained (extrinsic or intrinsic motivators) and the effort (physical or cognitive) required to acquire it, with a certain behavior being executed if the reward subjectively is evaluated to outweigh the effort.^32^ Research has revealed that the ventromedial prefrontal cortex signals reward value, whereas the dorsal anterior cingulate cortex integrates reward and effort to select a response.^33^ Upregulation of the dopaminergic system results in rewards being valued higher and efforts being valued lower.^32^, ^34^, ^35^ These findings are in line with studies showing that depletion of mesolimbic dopaminergic pathways is correlated with apathy in PD.^36^, ^37^, ^38^ Moreover, dopamine agonists (DA), which have a high affinity for D3 receptors that play a crucial role in the ventral reward system, are the main risk factors for ICD in PD.^39^

Consistent with this, reward sensitivity in effort‐based decision‐making is reduced in PD patients with apathy and PD patients off their dopaminergic medication,^40^, ^41^ and dopaminergic treatment leads to an increase in the willingness to exert effort in PD.^41^ Furthermore, a study recording the neural activity of the STN in PD patients identified a modulation in the low‐frequency range to reward and effort cues.^42^ These responses increased with higher rewards and were predictive of the acceptance of the presented offers. Another study examining effort‐based decision‐making ON and OFF STN‐DBS in a small sample of patients showed that STN‐DBS leads to faster responses, as well as a reduced sensitivity to both effort and reward.^35^ Altogether, these investigations implicate a crucial role of the STN and dopaminergic medication in effort‐based decision‐making. However, to date, such decision‐making has not been studied in PD patients treated with STN‐DBS over a long duration (chronic setting), and the impact of ICD on effort‐based decision‐making remains to be established. The aim of the present study was therefore to investigate the effect of chronic STN‐DBS and ICD on this form of decision‐making.

## Patients and Methods

### Ethical Approval

This study was approved by the local ethics committee (KEK Bern 2020‐01777) and was conducted in accordance with the Declaration of Helsinki. Written consent was obtained from all participants.

### Participants

Forty‐five patients with a diagnosis of idiopathic PD were recruited from the Movement Disorders Centre at the University Hospital of Bern, Switzerland. Ten patients (group 1) were scheduled for STN‐DBS, and 35 patients (group 2) were already treated with STN‐DBS. Surgical procedures and selection criteria for STN‐DBS in this hospital have previously been described in detail.^43^ Additional inclusion criteria for the study other than STN‐DBS and diagnosis of idiopathic PD were age 18 to 75 years, proficiency in French or German, absence of dementia (ruled out by detailed neuropsychological assessment and a minimum score of 24 in the Montreal Cognitive Assessment [MoCA]), absence of other major neurological or psychiatric illnesses, ability to provide informed consent, and no participation in pharmacological studies.

Patients of group 1 underwent the experimental procedures before and 4.4 (±0.4) months after STN‐DBS. In case of motor fluctuations during the experimental procedures, the assessment was paused until the *on* medication condition was reestablished. The time point of the postoperative visit was chosen so that inpatient optimization of STN‐DBS treatment, which was conducted 4 months after the operation as part of the clinical routine, had been finalized before the visit.

Group 2 underwent the experimental procedures on chronic STN‐DBS at an average of 40 (±31.2) months after surgery, with none of the participants tested less than 4 months after the surgery. Patients were assessed on their usual medication *on* and stimulation ON states. Two participants of group 2 had to be excluded from data analysis due to somnolence during the task in 1 participant and technical problems with the effort‐based decision‐making task in the other participant.

## Neurological, Psychiatric, and Cognitive Measures

Neurological assessments included the Movement Disorders Society Unified Parkinson's Disease Rating Scale (MDS‐UPDRS),^44^ with Part III of the scale assessed in the usual medication *on* (and postoperatively medication *on* plus stimulation ON) condition. Based on the reported dopaminergic medication, levodopa equivalent daily dose (LEDD) and DA daily dose were calculated according to published guidelines.^45^, ^46^

Neuropsychiatric evaluation entailed the Questionnaire for Impulsive‐Compulsive Disorders in Parkinson's Disease Rating Scale (QUIP‐RS) present version and anytime version,^47^ the Hospital Anxiety and Depression Scale,^48^ and the Starkstein Apathy Scale.^49^ Furthermore, to diagnose apathy, the diagnostic criteria for apathy established by an expert consensus in 2018^50^ were applied. All these scales are recommended by the Movement Disorders Society to assess neuropsychiatric symptoms in PD.^51^, ^52^

Cognitive testing included the MoCA,^53^ digit span forward and backward,^54^ phonemic and semantic verbal fluency,^55^, ^56^ modified Trail Making Test Parts A and B (TMT A and B),^57^ symbol‐search task,^58^ and the Stroop color‐word interference test.^59^

### Effort‐Based Decision‐Making Task

The apple tree task developed by the research team of Prof. Husain^40^, ^41^ was used to measure effort‐based decision‐making. Participants were seated in front of a laptop computer with a 17‐inch screen, with 2 handheld dynamometers (Baseline, standard 200‐lb capacity) placed in front of them. The dynamometers were attached to a metal frame with wires and automatic suspension so that participants did not have to hold them between responses. The experimental task was programmed in MATLAB^60^ and implemented with Unity.^61^ At the beginning of the experiment, participants were asked to squeeze the dynamometer with their dominant hand as strongly as possible thrice. The maximum force obtained was then defined as the maximum voluntary grip force (MVC). In the following experiment, all responses were normalized to this MVC.

**FIG. 1:**
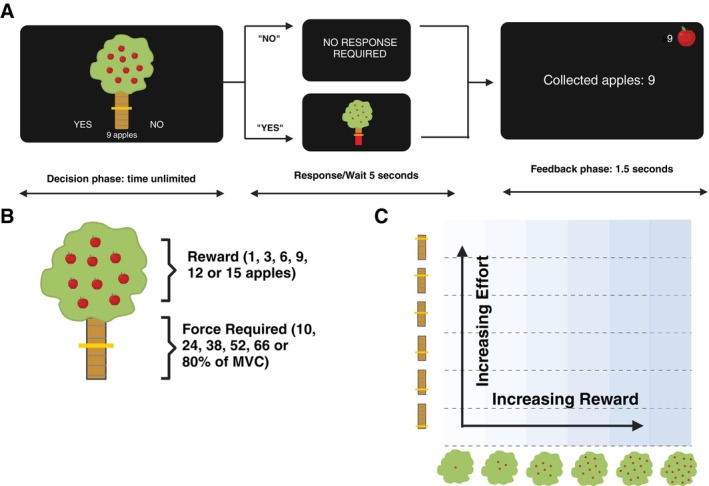
(**A**) Example of a trial used in the effort‐based decision‐making task. (**B**) Six levels of reward (displayed as apples) and effort (indicated on the tree trunk, adjusted to maximum voluntary contraction) were combined, (**C**) resulting in 36 trials per block.

During the experiment, participants were presented with offers, visualized on an apple tree (Fig. 1). Offers included different levels of reward (depicted as 1, 3, 6, 9, 12, or 15 apples; each worth the equivalent to 1 Swiss Rappen = ~1 US cent) in return for different levels of effort (indicated by a yellow bar on the tree trunk, corresponding to 10, 24, 38, 52, 66, and 80% of the previously defined MVC). The 6 reward and effort levels were systematically combined, resulting in 36 conditions. Each of 5 blocks consisted of those 36 conditions, which were pseudorandomized within each block, amounting to 180 trials. All participants were presented with the offers in the same order.

Each block started with a “decision phase,” during which the participant was presented with 36 different offers and had to decide whether the reward (number of apples) was worth the effort (yellow bar on the tree trunk). They had to shortly squeeze the controller on the left to accept an offer (“yes”) and the controller on the right to decline (“no”). In the “apple picking phase,” patients had to pick the apples of accepted offers by squeezing the dynamometer to the required force for at least 2 seconds. If they were successful, the collected apples were added to a counter on the top right of the screen. For failed or rejected offers, no apples were gained, but no apples were deducted either. During the rejected trials, participants could relax for 5 seconds to avoid temporal discounting. Additionally, to avoid a tiring effect, 25% of accepted offers did not require a grip force (participants were instructed about this beforehand). In these cases, which were pseudorandomized across all trials, participants were informed by a message on the screen that they could relax instead of picking apples. Participants were instructed that they could use their hands interchangeably during the experiment to avoid tiring but that only 1 hand at a time should be used.

Before the first block, participants were presented with 12 practice trials. During the instruction, the participants were informed about the value of each apple and that they would be paid the gains at the end of the experiment in the form of a supermarket voucher (rounded up, at the end of the experiment, to 10 or 20 Swiss francs, equivalent to $10 or $20). Performance feedback was given during the experiment on the top left of the screen and after each block. For each trial, acceptance (yes, no), decision time, and grip force (sampled at 500 Hz) were recorded.

### Statistical Analysis

R (version 4.3.3)^62^ was used to analyze data. Means and standard deviations were calculated for neurological, neuropsychiatric, and cognitive measures. For comparisons between dependent variables (eg pre‐ and postoperative comparison in group 1), paired sample *t* tests were used for normally distributed data and paired sample Wilcoxon tests for nonnormally distributed data. For comparisons between independent variables (eg ICD vs. no ICD comparisons in group 2), *t* tests were used for normally distributed data and Mann‐Whitney *U* tests for nonnormally distributed data.

Regarding the analysis of the effort‐based decision‐making task, data were inspected for missing values that could result from accidental squeezes (1.8% of the data were missing) and were imputed using a random forest imputation algorithm.^63^ To visualize data, heat maps showing the proportion of accepted offers for each reward and effort combination were drawn for group 1 preoperative measurements, group 1 postoperative measurements, and group 2 measurements. The mean acceptance rate over all trials was calculated for each visit. Furthermore, mean acceptance rates were calculated separately for each reward and effort level. Mean reaction times across all trials were calculated for each patient visit. Reaction times of >8 were identified as outliners and were not included in the calculation of the mean reaction times. To visualize data for mean acceptance rates and reaction time, box plots were used. Linear mixed‐effects models^64^, ^65^ were used on the combined dataset of groups 1 and 2 to assess the impact of different variables on acceptance rates and reaction times.

## Results

Baseline variables and postoperative outcomes for group 1 are summarized in Table 1. In group 1, 7 (70%) participants had at least 1 ICD as classified by QUIP‐RS above a cutoff before STN‐DBS compared to 5 (50%) participants at follow‐up. The baseline variables for group 2 are summarized in Table 2. Of the 33 participants in group 2, 17 (52%) had at least 1 ICD according to the QUIP‐RS present version. Information on stimulation parameters is presented in Table S4.

**TABLE 1:** Demographic data and pre‐ and postoperative neurological, neuropsychiatric, and cognitive measures of group 1

	Baseline	4‐Month follow‐up	*P*‐value
n	10	10	n/a
Age (yr)	57.8 (±8.1)	n/a	n/a
Gender (% female)	3/7 (30%)	n/a	n/a
Disease duration (yr)	8.1 (±3.4)	n/a	n/a
Months since DBS surgery	n/a	4.4 (±0.4)	n/a
MDS‐UPDRS I	14.1 (±3.2)	6.8 (±3.9)	<0.001***
MDS‐UPDRS II	15.6 (±5.6)	5.6 (±4.9)	<0.001***
MDS‐UPDRS III medication *on* (stimulation ON)	23.8 (±11.1)	18.6 (±12.0)	0.206
MDS‐UPDRS IV	8.7 (±3.3)	1.9 (±2.5)	0.007**^,^ª
LEDD (mg/day)	1413.0 (±534.6)	610.0 (±329.0)	<0.001***
DA daily dose (mg/day)	175.0 (±147.4)	74.0 (±49.7)	0.050ª
Starkstein Apathy Scale	9.2 (±2.9)	10.6 (±3.7)	0.336
Apathy diagnosis	0 (0%)	0 (0%)	n/a
HADS anxiety	7.8 (±3.9)	4.4 (±1.6)	0.057ª
HADS depression	6.8 (±4.2)	2.1 (±1.9)	0.022*^,^ª
QUIP‐RS present	14.8 (±13.0)	8.6 (±8.9)	0.033*^,^ª
ICD according to QUIP‐RS present	7/10 (70%)	5/10 (50%)	n/a
QUIP‐RS anytime	18.5 (±14.5)	21.3 (±18.4)	0.515
MoCA	27.8 (±1.7)	27.9 (±1.5)	0.872
Phonemic verbal fluency	−0.2 (±0.7)	−0.7 (±0.5)	0.047*
Semantic verbal fluency	−0.2 (±0.8)	−1.0 (±0.6)	0.027*^,^ª
TMT A	−0.4 (±1.3)	−0.7 (±1.1)	0.626
TMT B	−1.1 (±1.1)	−1.4 (±0.7)	0.413
Digit span forward	0.0 (±0.6)	−0.0 (±0.8)	0.944ª
Digit span backward	−0.2 (±0.7)	−0.1 (±1.0)	0.960
Symbol search	−0.5 (±0.7)	−0.8 (±0.4)	0.242
Stroop color naming time	0.1 (±0.9)	−0.8 (±0.8)	0.010*
Stroop inhibition time	−0.3 (±0.5)	−1.2 (±1.1)	0.013*^,^ª
Stroop inhibition errors	0.0 (±1.0)	−0.4 (±0.9)	0.313ª

*Notes*: Reported values are means (±standard deviation) for numeric variables and counts (% percentages) for categorical variables. Cognitive tests except for MoCA are reported in standardized *z* values (±standard deviation). *P*‐values are paired *t* tests for normally distributed variables and Wilcoxon signed‐rank tests for nonnormally distributed data (indicated by superscript letter “a”). *P*‐values of <0.05 are considered as significant (*p = <0.05, **p = <0.01, ***p= <0.001); no multiple comparison correction was made. n/a = not applicable.

Abbreviations: DBS, deep brain stimulation; MDS‐UPDRS, Movement Disorder's Society Unified Parkinson's Disease Rating Scale; LEDD, levodopa equivalent daily dose; DA daily dose, dopamine agonist daily dose; apathy diagnosis, apathy according to the diagnostic criteria of Robert et al. 2018; HADS, Hospital Anxiety and Depression Scale; QUIP‐RS, Questionnaire for Impulsive‐Compulsive Disorders in Parkinson's Disease Rating Scale; ICD, impulse control disorder; ICD according to QUIP‐RS present, at least 1 of the items of the QUIP‐RS above cutoff; MoCA, Montreal Cognitive Assessment; TMT, Trail Making Test.

**TABLE 2:** Demographic data and neurological, neuropsychiatric, and cognitive measures of group 2

	Overall	Without ICD	With ICD	*P*‐value
N	33	17	16	n/a
Age (yr)	60.9 (±7.8)	60.9 (±8.1)	60.9 (±7.8)	0.984
Gender (% female)	10 (30.3%)	4 (23.5%)	6 (37.5%)	n/a
Disease duration (yr)	13.6 (±6.3)	11.8 (±6.5)	15.6 (±5.7)	0.036*^,^ª
Months since DBS surgery	40.0 (±31.2)	35.6 (±32.0)	44.6 (±30.7)	0.494ª
MDS‐UPDRS I	9.1 (±5.2)	7.2 (±4.6)	11.1 (±5.2)	0.018*^,^ª
MDS‐UPDRS II	9.8 (±5.8)	7.1 (±4.8)	12.6 (±5.5)	0.005**
MDS‐UPDRS III medication *on* (stimulation ON)	16.0 (±9.4)	14.5 (±10.3)	17.6 (±8.3)	0.134ª
MDS‐UPDRS IV	4.0 (±4.2)	2.6 (±3.3)	5.4 (±4.7)	0.063ª
LEDD (mg/day)	541.7 (±298.4)	400.4 (±222.0)	691.9 (±301.2)	0.004**
DA daily dose (mg/day)	129.8 (±102.3)	90.4 (±81.0)	171.6 (±108.2)	0.019*^,^ª
Starkstein Apathy Scale	12.3 (±4.7)	13.4 (±5.3)	11.2 (±3.8)	0.270ª
Apathy diagnosis	7 (21.2%)	7 (41.2%)	0 (0%)	n/a
HADS anxiety	4.1 (±3.4)	2.8 (±2.4)	5.4 (±3.7)	0.035*^,^ª
HADS depression	3.7 (±2.7)	3.1 (±3.0)	4.3 (±2.1)	0.069ª
QUIP‐RS present	11.6 (±15.4)	1.4 (±2.2)	22.4 (±16.1)	<0.001***^,^ª
QUIP‐RS anytime	21.5 (±18.4)	13.5 (±15.2)	30.1 (±18.1)	0.007**^,^ª
MoCA	27.6 (±1.8)	27.7 (±2.0)	27.5 (±1.6)	0.475ª
Phonemic verbal fluency	−0.0 (±0.9)	−0.2 (±0.7)	0.1 (±1.0)	0.394
Semantic verbal fluency	−0.1 (±1.0)	−0.3 (±1.0)	0.0 (±1.0)	0.431
TMT A	−0.4 (±0.8)	−0.4 (0.6)	−0.5 (1.0)	0.299ª
TMT B	−0.9 (±1.0)	−1.2 (±0.8)	−0.6 (±1.0)	0.080
Digit span forward	0.5 (±1.0)	0.5 (±1.0)	0.4 (±0.9)	0.734
Digit span backward	−0.5 (±0.9)	−0.6 (±0.8)	−0.4 (±1.1)	0.727
Symbol search	−0.5 (±0.6)	−0.5 (±0.8)	−0.5 (±0.4)	0.959
Stroop color naming time	−0.5 (±1.0)	−0.8 (±1.0)	−0.3 (±0.9)	0.176
Stroop inhibition time	0.1 (±0.9)	−0.2 (±0.9)	0.3 (±0.8)	0.091ª
Stroop inhibition errors	−0.1 (±1.0)	0.1 (±1.0)	0.3 (±1.0)	0.209ª

*Notes*: Reported values are means (±standard deviation) for numeric variables and counts (% percentages) for categorical variables. Cognitive tests except for MoCA are reported in standardized *z* values (±standard deviation). *P*‐values are independent *t* tests for normally distributed variables and independent Wilcoxon tests for nonnormally distributed data (indicated by superscript letter “a”). *P*‐values of <0.05 are considered as significant (*p = <0.05, **p = <0.01, ***p = <0.001); no multiple comparison correction was made. n/a = not applicable.

Abbreviations: ICD, Impulse control disorder; DBS, deep brain stimulation; MDS‐UPDRS, Movement Disorder's Society Unified Parkinson's Disease Rating Scale; LEDD, levodopa equivalent daily dose; DA daily dose, dopamine agonist daily dose; apathy diagnosis, apathy according to the diagnostic criteria of Robert et al. 2018; HADS, Hospital Anxiety and Depression Scale; QUIP‐RS, Questionnaire for Impulsive‐Compulsive Disorders in Parkinson's Disease Rating Scale; MoCA, Montreal Cognitive Assessment; TMT, Trail Making Test.

### Mean Acceptance Rates in Effort‐Based Decision‐Making

**FIG. 2:**
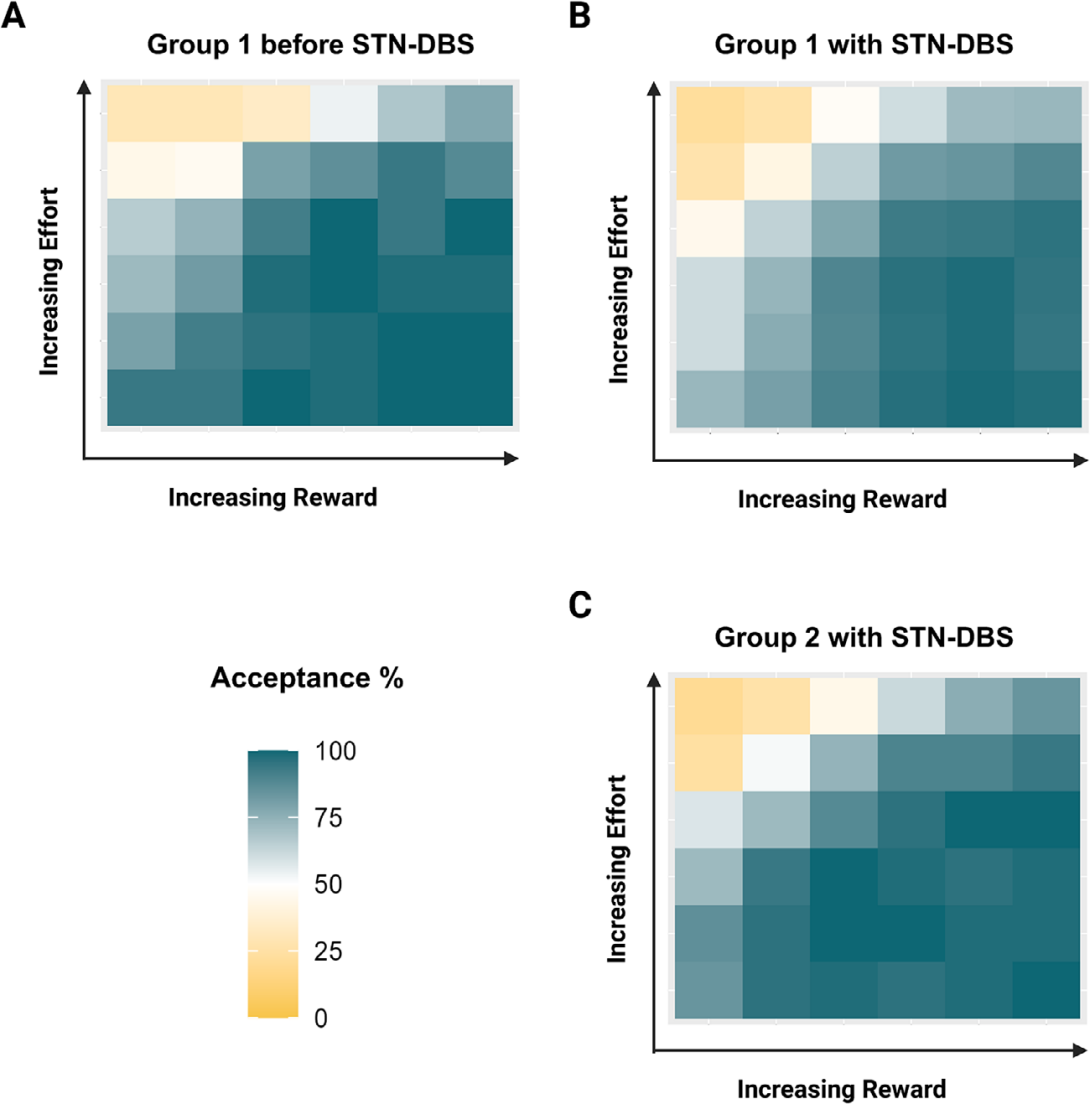
Heat maps display the acceptance rate for (**A**) group 1 before STN‐DBS (subthalamic nucleus deep brain stimulation), (**B**) group 1 with STN‐DBS after 4 months, and (**C**) group 2 with chronic STN‐DBS. Each cell of the heat map represents 1 of the 36 combinations of reward and effort.

**FIG. 3:**
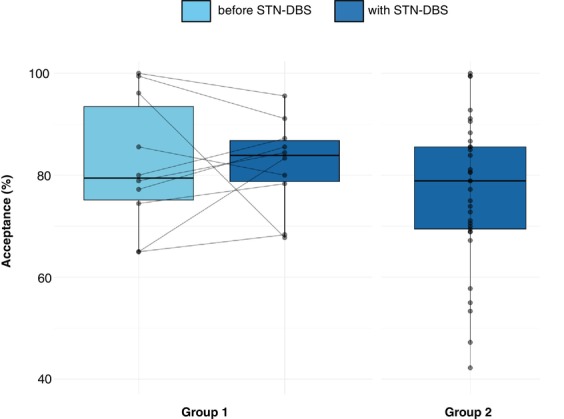
Mean acceptance rates across all trials of the effort‐based decision‐making task before STN‐DBS (subthalamic nucleus deep brain stimulation (group 1, light blue) and with STN‐DBS (groups 1 and 2, dark blue).

Across all measurements, acceptance rate in the effort‐based decision‐making task significantly increased with reward (*χ* ^2^ = 1180.3, *P* ≤ 0.001) and decreased with effort (*χ* ^2^ = 1254.8, *P* ≤ 0.001). Mean acceptance rate across all trials did not differ pre‐ and postoperatively in group 1 (82.2% [±13.0] vs. 82.2% [±8.9], *P* > 0.999). There was also no difference in mean acceptance rate across all trials between postoperative measurements of groups 1 and 2 (82.2% [±8.9] vs. 76.6% [±14.4], *P* = 0.156) (Figs. 2 and 3).

For the overall dataset (groups 1 and 2 combined), no significant predictors for mean acceptance rate were identified in a mixed linear model, including LEDD, MDS‐UPDRS Part III, MoCA, STN‐DBS (yes/no), and ICD (yes/no) as fixed effects and subject ID as random effect (Table S1).

### Acceptance Rates at Different Reward Levels for ICD versus No ICD

To assess the relationship between each reward level and the presence of ICD, a mixed‐effects model was used. The model included acceptance rates as the outcome variable, with fixed effects for the categorical variable reward level (levels 1–6), ICD (yes/no), and their interaction, along with additional covariates LEDD, MDS‐UPDRS Part III, STN‐DBS (yes/no), and MoCA, and a random intercept for subject ID. Results revealed that acceptance rate increased with each reward level when all other variables were kept at 0, but no significant main or interaction effects for any other predictors were found (Table 3).

**TABLE 3:** Predictors for acceptance rates and reaction times

Predictors for acceptance rates at different reward levels			
Variable	Estimate	Standard error	*P*‐value
Intercept	42.53	28.68	0.141
Reward level 2	12.53	3.71	0.001***
Reward level 3	25.20	3.71	<0.001***
Reward level 4	33.07	3.71	<0.001***
Reward level 5	34.53	3.71	<0.001***
Reward level 6	35.20	3.71	<0.001***
ICD (yes)	−2.81	4.68	0.548
Reward level 2 × ICD	−2.18	5.11	0.671
Reward level 3 × ICD	1.23	5.11	0.810
Reward level 4 × ICD	2.29	5.11	0.654
Reward level 5 × ICD	4.99	5.11	0.330
Reward level 6 × ICD	5.28	5.11	0.303
LEDD (mg/day)	−0.00	0.01	0.461
MDS‐UPDRS Part III	0.12	0.18	0.507
DBS (yes)	−3.35	4.39	0.446
MoCA	0.58	0.97	0.553

Predictors for acceptance rates at different effort levels			
Variable	Estimate	Standard error	*P*‐value
Intercept	81.49	29.24	0.006**
Effort level 2	−2.00	3.90	0.609
Effort level 3	−2.13	3.90	0.585
Effort level 4	−11.07	3.90	0.005**
Effort level 5	−30.00	3.90	<0.001***
Effort level 6	−48.67	3.90	<0.001***
ICD (yes)	−4.94	4.86	0.311
Effort level 2 × ICD	−0.98	5.37	0.856
Effort level 3 × ICD	−2.99	5.37	0.579
Effort level 4 × ICD	1.42	5.37	0.791
Effort level 5 × ICD	11.31	5.37	0.036*
Effort level 6 × ICD	14.38	5.37	0.008**
LEDD (mg/day)	−0.00	0.01	0.524
MDS‐UPDRS Part III	0.11	0.18	0.565
DBS (yes)	−3.17	4.53	0.485
MoCA	0.58	0.99	0.561

Predictors for overall reaction times			
Variable	Estimate	Standard error	*P*‐value
Intercept	1.49	0.61	0.020
Age (yr)	0.01	0.01	0.458
MDS‐UPDRS Part III	0.01	0.01	0.057
STN‐DBS (yes)	−0.33	0.10	0.012*
ICD (yes)	−0.24	0.11	0.046*
Symbol‐search *z* score	−0.28	0.09	0.007**

*Notes*: Mixed‐effects model with acceptance rates in %, respectively overall reaction times, as outcome; fixed effects variables listed earlier; subject ID as random effect.

Abbreviations: ICD, impulse control disorder; LEDD, levodopa equivalent daily dose; MDS‐UPDRS, Movement Disorder's Society Unified Parkinson's Disease Rating Scale; DBS, deep brain stimulation; STN‐DBS, subthalamic nucleus deep brain stimulation; MoCA, Montreal Cognitive Assessment.

### Acceptance Rates at Different Effort Levels for ICD versus No ICD

**FIG. 4:**
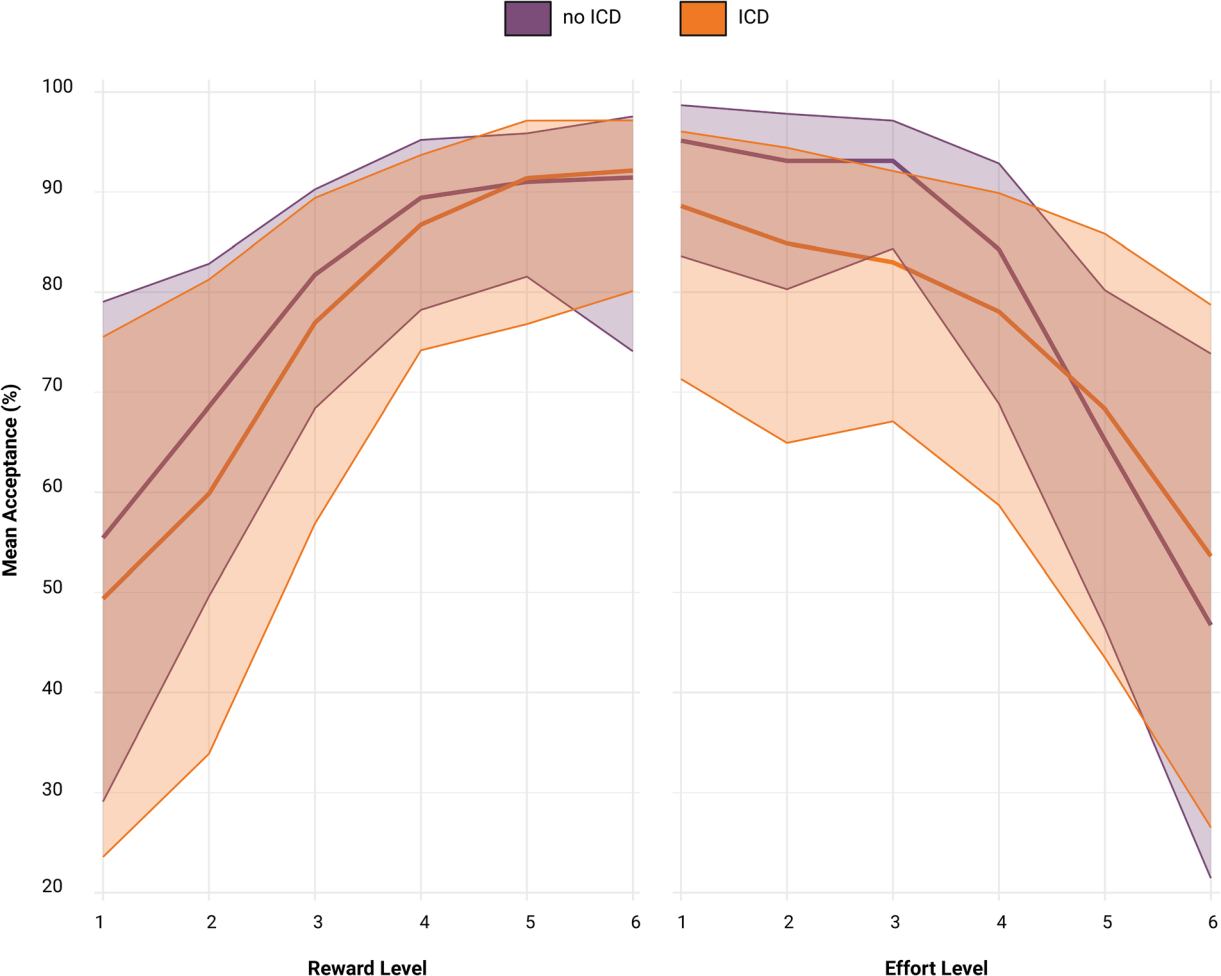
Results from overall dataset (all measurements of groups 1 and 2 combined). Means and 95% logit‐transformed confidence intervals for acceptance rate at each of the 6 reward and effort levels, stratified by impulse control disorder.

Similarly, to assess the relationship between each effort level and the presence of ICD, a mixed‐effects model was used for the whole dataset (groups 1 and 2 combined). The model included acceptance rates as the outcome variable, with fixed effects for effort level, ICD (yes/no), and their interaction, along with additional covariates LEDD, MDS‐UPDRS Part III, STN‐DBS (yes/no), and MoCA, and a random intercept for subject ID. Results revealed significantly lower acceptance rates for effort levels 4, 5, and 6 compared to effort level 1 when all other variables were kept at 0. Furthermore, significant interaction terms revealed that participants with ICD were more likely to accept offers at the high effort levels 5 and 6 (Fig. 4; Table 3).

### Factors Influencing Reaction Times in Effort‐Based Decision‐Making

**FIG. 5:**
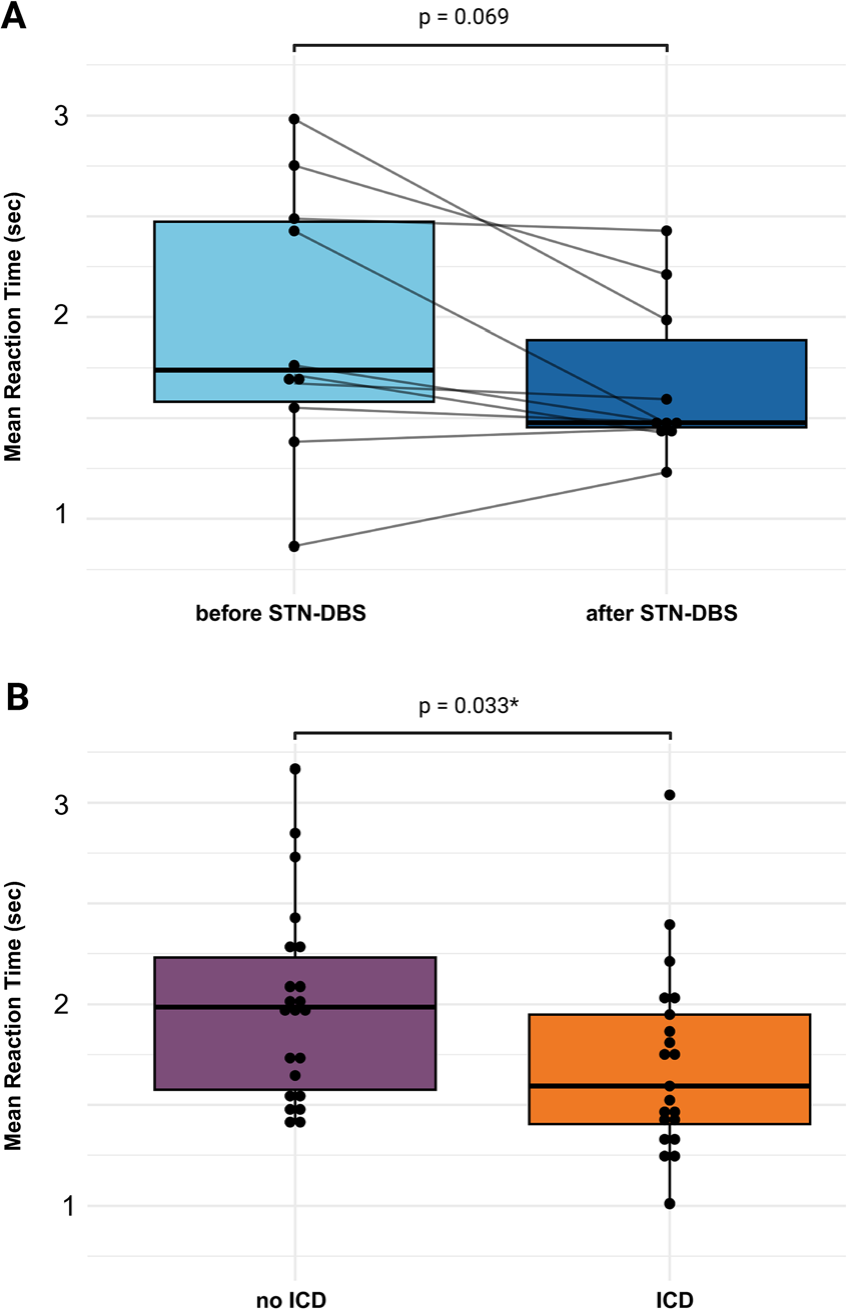
Mean reaction time (in seconds) across all effort‐based decision‐making trials. (**A**) Comparison of reaction time in group 1 before and 4 months after STN‐DBS (subthalamic nucleus deep brain stimulation). (**B**) Reaction time stratified by ICD (impulse control disorder) for patients with STN‐DBS (groups 1 and 2 combined).

In group 1, decision time was slower before STN‐DBS than after STN‐DBS (1.96 s [±0.67] vs. 1.67 s [±0.39], *P* = 0.069) (Fig. 5). In participants with STN‐DBS (groups 1 and 2 combined, without preoperative data), reaction times were faster in ICD versus no ICD (1.71 sec [±0.47] vs. 1.99 sec [±0.48], *P* = 0.033*) (Fig. 5).

To identify the predictors of reaction times in the overall dataset (groups 1 and 2 combined, including preoperative data), a mixed linear effects model with reaction times as outcome variable; age, MDS‐UPDRS Part III, STN‐DBS (yes/no), LEDD, ICD (yes/no), symbol search, TMT A, and Stroop inhibition time as fixed effects; and subject ID as random effect were fitted (Table S2). This initial model explained 89.5% of the variance in reaction times, with symbol search and ICD as significant predictors. However, there was collinearity between MDS‐UPDRS Part III and TMT A (correlation of fixed effects = 0.429), Stroop inhibition time and symbol search (correlation of fixed effects = −0.474), and LEDD and STN‐DBS (correlation of fixed effects = 0.734) (Table S3).

Because of collinearity, LEDD, TMT A, and Stroop inhibition time, which were all not significant predictors, were excluded from the model. In the adjusted model with age, MDS‐UPDRS Part III, DBS, ICD (yes/no), and symbol search, all fixed effects contributed significantly to the model (Table 3), with STN‐DBS reducing reaction times on average by 0.33 s [±0.07] (*P* = 0.003**), symbol search reducing reaction time by 0.28 s [±0.07] per standard deviation increase (*P* = 0.003**), ICD reducing reaction time by 0.26 s [±0.09] (*P* = 0.020), and MDS‐UPDRS Part III increasing reaction time by 0.01 s [±0.00] per point increase (*P* = 0.026*). The final model including subject ID as a random effect explained 95.3% of the variance in reaction times.

## Discussion

This study demonstrates that both STN‐DBS and ICD lead to faster decision times in an effort‐based decision‐making task, and that ICD increases the willingness to exert high levels of effort. Importantly, these effects remain significant even when controlling for motor symptom burden, dopaminergic medication, and cognitive measures.

The higher acceptance rate for high‐effort trials associated with ICD may reflect either impulsive decision‐making or a positive increase in motivated behavior. In the past, ICD in PD has been associated with reduced learning from punishment and risk‐taking behavior,^66^, ^67^ but motor inhibition seems to be unaffected or even improved in patients with ICD.^68^, ^69^ In the present sample with STN‐DBS (group 2), ICD patients did not differ from patients without ICD on a range of cognitive tasks, including Stroop error rate, showing the importance of using specific tasks examining decision‐making in clinical practice. ICD remained a significant predictor of faster reaction time even when controlling for cognitive processing speed, motor symptom burden, dopaminergic medication dosage, and STN‐DBS. Furthermore, compared to healthy controls performing the same effort‐based decision‐making task, participants with ICD in this sample did not accept more offers or react faster.^40^ Thus, the higher acceptance rate in high‐effort trials has to be interpreted as a positive increase in motivated behavior rather than impulsive choice. In the present study, most of the participants had mild ICD. Because ICD has to be understood as a spectrum rather than a category, positive effects on cognition and motivated behavior may be observed in mild ICD, whereas impulsivity and other dysexecutive symptoms may occur in more severe ICD.

The observation that STN‐DBS speeds up decision‐making is in line with studies showing that turning stimulation ON versus OFF leads to faster responses during effort‐based decision‐making and tasks involving competing choices.^16^, ^17^, ^35^ In healthy individuals, the STN acts like a “brake,” inhibiting or pausing predominant motor responses via a hyper‐direct pathway.^70^, ^71^ STN‐DBS can have both positive and negative effects on motor inhibitory control, depending on the exact stimulation site.^18^, ^19^, ^20^, ^21^, ^22^ Reaction times under STN‐DBS are comparable to healthy controls,^40^ and unlike in other studies,^18^, ^19^ the error rate in the Stroop task did not increase postoperatively in our sample. Thus, the faster reaction times compared to participants without STN‐DBS cannot be attributed simply to motor impulsivity and may rather reflect facilitation in decision‐making per se.

In terms of behavioral outcomes, a tendency for improvement in ICD and depressive symptoms, but worsening of language tasks involving speed, was observed in the present study. These results align with some prior research showing a decrease in these symptoms^9^, ^72^, ^73^, ^74^ and a recent guideline recommending STN‐DBS as a second‐line treatment strategy for ICD.^75^ However, because these results would not withstand multiple comparison corrections, they have to be interpreted with caution. Other studies showed opposite effects with new‐onset ICD or worsening of ICD in a subpopulation of patients remaining on higher doses of dopaminergic treatment after DBS.^11^ The observation that apathy scores remained unaffected by STN‐DBS is not consistent, with recent meta‐analyses demonstrating an increase in apathy after STN‐DBS.^13^, ^14^ However, the low prevalence of apathy might be explained by different factors.

First, a recent study has shown that the main driver for postoperative apathy is cognitive impairment,^76^ but for the present study, only PD patients with no or mild cognitive impairment were recruited. Furthermore, a study with an especially marked postoperative decrease in dopaminergic medication to study the pathophysiology of postoperative apathy could indeed show increased postoperative apathy, correlating with more severe mesolimbic dopaminergic denervation.^36^ In that same cohort, the reintroduction of a DA reversed apathy in a randomized controlled trial.^77^ In the present study, patients were closely followed and treated with a combination of small but frequent doses of levodopa, together with individually titrated DAs to prevent postoperative apathy. This might explain not only the lack of apathy that was observed but also the relatively high prevalence of mild ICD. Altogether, this highlights the importance of postoperative clinical management of neuropsychiatric symptoms, evaluated longitudinally as part of routine clinical practice, with individualized stimulation amplitudes and medication to balance neuropsychiatric symptoms. Last but not least, the fact that no postoperative increase in apathy was observed in the present study might be due to the relatively short time frame of follow‐up. Indeed, a previous study showed that ICD decreased and apathy increased gradually over several months after STN‐DBS as a result of gradual desensitization of the dopaminergic system.^78^ Interestingly, none of the participants with ICD in the present sample fulfilled the criteria for apathy, despite some earlier studies showing a co‐occurrence of impulsivity and apathy.^79^ Again, this might be explained by the fact that participants were cognitively relatively unimpaired, as the co‐occurrence of impulsivity and apathy has been linked to dysexecutive symptoms^80^ and thus would be expected to occur more typically in cognitive rather than motivational apathy.^76^

Limitations of this study include a lack of information regarding the exact stimulation site within the STN (eg, volume of tissue activated), a relatively low number of preoperative assessments in group 1, and as previously stated, a lack of severity definition for ICD. Furthermore, with effort‐based decision‐making, only 1 possible contributor to ICD was examined in an experimental situation, whereas other underlying pathological mechanisms and aspects of impulsivity were not assessed. However, the strengths of this study are the naturalistic setting, as well as the detailed neuropsychiatric and cognitive assessments.

In conclusion, this study reveals that in PD patients, ICD enhances the willingness to produce high levels of effort to obtain rewards, which most likely reflects an increase in motivated behavior. Furthermore, both STN‐DBS and ICD facilitate effort‐based decision‐making by decreasing decision time. In the clinical setting, stimulation and dopaminergic medication should be titrated carefully to control motor symptoms and to balance the spectrum of neuropsychiatric symptoms. In this context, using decision‐making tasks in addition to the commonly used self‐report scales may be useful, because they can capture subtle changes more objectively.

## Author Roles

1. Research project: A. Conception, B. Organization, C. Execution, 2. Statistical analysis: A. Design, B. Execution, C. Review and critique; 3. Manuscript preparation: A. Writing of first draft, B. Review and critique.

D.A.: 1A, 1B, 1C, 2A, 2B, 3A

K.P.: 2A, 2B, 2C, 3B

M.S.: 2A, 2B, 2C, 3B

I.D.: 1B, 2C, 3B

M.E.M.‐G.: 1B, 2C, 3B

L.C.B.: 1B, 1C, 2C, 3B

A.D.M.: 2C, 3B

G.T.: 2C, 3B

A.D.: 2C, 3B

J.W.: 2C, 3B

L.M. L.: 2C, 3B

C.P.: 2C, 3B

D.C.: 1A, 1B, 2C, 3B

T.N.: 1A, 1B, 2C, 3B

M.H.: 1B, 2C, 3B

P.K.: 1A, 2A, 3A, 2C, 3A, 3B

## Disclosures

**Ethical Compliance Statement:** This study was approved by the local ethics committee (KEK Bern 2020‐01777) and was conducted in accordance with the Declaration of Helsinki. Written consent was obtained from all participants. All the authors have read and complied with the journal's position on issues involved in ethical publication and affirm that this work is consistent with those guidelines.

**Funding Sources and Conflicts of Interest:** This project was financed by a research grant from the Swiss Parkinson Association, to D.A. There are no conflicts of interest or other funding sources in relation to the present work.

**Financial Disclosures for the Previous 12 Months:** D.A. has received a research grant from the Swiss Parkinson Association in relation to the present work. M.S. recieved a research grant from Gottfried and Julia Bangerter‐Rhyner‐Stiftung paid through his institution, and financial support from Boston Scientic, Medtronic, Zambon and Bial not related to the present word. I.D.recieved research grants from Boston Scientific and the Swiss National Science Foundation, as well as travel reimbursement and support for raising awareness in PD (video project) from Zambon, AbbVie, Boston Scientific, Bial, Ever, UCB, Medtronic, Spirig, Merz, and GE Healthcare; and has also served on the advisory boards of Ever, Spirig, and AbbVie. A.D.M. recieved a research grant by Boston Scientific outside the submitted work. G.T. received financial support from Boston Scientific and Medtronic not related to the present work. Research agreement with RuneLabs is not related to the present work. A. D. reports sponsored travel and referee fees from AbbVie and Bial, both outside the submitted work. J.W. recieved travel grants from Merz and lecture fees from Abbvie, both outside the submitted work. L.M.L. recieved a research grant from the Jacques and Gloria Gossweiler Foundation outside the submitted work and has served on the advisory board of Bial. C.P. is the co‐founder of Aleva Neurotherapeutics, a medical device company based in Lausanne Switzerland, currently without any active role in the company. D. C. recieved research grants from the Swiss Heart Foundation and the ETH RESC Suva Funding Programme, both outside the submitted work. M. H. recieved funding from the Wellcome Trust and the NIHR Oxford Health Biomedical Research Centre, both outside the submitted work. P.K. reports research or educational grants from the Swiss National Science Foundation (FNS 323530_177577/FNS 32003BL_197709‐1/FNS 33IC30_198772), ROGER DE SPOELBERCH Foundation, Fondation Louis‐Jeantet, Carigest, Institut National de la Santé et de la Recherche Médicale, France Parkinson, Edmond Safra Philanthropic Foundation, Bertarelli Foundation, Annemarie Opprecht Foundation, Parkinson Schweiz, the Michael J. Fox Foundation, Aleva Neurotherapeutics, Boston Scientific, Medtronic, St. Jude Medical, GE Healthcare, Idorsia, and UCB, all paid to employing institutions; lecturing fees from employing institutions such as from Boston Scientific, Bial, and Advisis; and travel expenses to scientific meetings from Boston Scientific, Zambon, AbbVie, and Merz Pharma (Schweiz) AG.

## Supporting information


**Table S1.** Mixed linear model for acceptance rate without effort or reward levels.
**Table S2.** Initial mixed linear model for reaction time.
**Table S3.** Initial mixed linear model for reaction time collinearity results.
**Table S4.** Average stimulation parameters for STN–DBS (subthalamic nucleus deep brain stimulation).

## Data Availability

The data that support the findings of this study are available on request from the corresponding author. The data are not publicly available due to privacy or ethical restrictions.
