# Supplementary material for: Impulse Control Disorders and Effort‐Based Decision‐Making in Parkinson's Disease Patients with Subthalamic Nucleus Deep Brain Stimulation
Source: Mov Disord Clin Pract. 2025 Jan 3;12(4):484–96. doi: 10.1002/mdc3.14318 (PMC11998690; doi:10.1002/mdc3.14318)
Supplement: Supplementary file 1 — Table S1. Mixed linear model for acceptance rate without effort or reward levels. Table S2. Initial mixed linear model for reaction time. Table S3. Initial mixed linear model for reaction time collinearity results. Table S4. Average stimulation parameters for STN–DBS (subthalamic nucleus deep brain stimulation). [file MDC3-12-484-s001.docx]

**Supplementary Material**

**Table 1 Supplementary Material.** Mixed linear model for acceptance rate without effort or reward levels.

| **Variable** | **Estimate** | **Std error** | **p-value** |
| --- | --- | --- | --- |
| **Intercept** | 67.32 | 34.2 | 0.055 |
| **LEDD (mg/day)** | 0.00 | 0.01 | 0.857 |
| **MDS-UPDRS part III** | 0.00 | 0.21 | 0.991 |
| **MoCA** | 0.49 | 1.17 | 0.678 |
| **STN-DBS (yes)** | -1.98 | 6.35 | 0.758 |
| **ICD (yes)** | -3.29 | 4.37 | 0.456 |

Mixed effects model with acceptance rate in % as outcome; fixed effects variables listed above; subject ID as random effect.

**Table 2 Supplementary Material.** Initial mixed linear model for reaction time.

| **Variable** | **Estimate** | **Std error** | **p-value** |
| --- | --- | --- | --- |
| **Intercept** | 1.39 | 0.65 | 0.039 |
| **Age** | 0.01 | 0.01 | 0.403 |
| **MDS-UPDRS part III** | 0.01 | 0.01 | 0.157 |
| **STN-DBS (yes)** | -0.28 | 0.18 | 0.164 |
| **LEDD (mg/day)** | -0.00 | 0.00 | 0.988 |
| **ICD (yes)** | -0.24 | 0.14 | 0.100 |
| **Symbol Search** | -0.30 | 0.11 | 0.015 |
| **TMT A** | -0.02 | 0.06 | 0.804 |
| **Stroop Inhibition Time** | 0.08 | 0.08 | 0.319 |

Mixed effects model with reaction time as outcome; fixed effects variables listed above; subject ID as random effect.

**Table 3 Supplementary Material.** Initial mixed linear model for reaction time collinearity results.

| **Variable** | **Intercept** | **Age** | **UPDRS III** | **STN-DBS** | **LEDD** | **ICD** | **Symbol search** | **TMT A** |
| --- | --- | --- | --- | --- | --- | --- | --- | --- |
| **Age** | -0.911 |  |  |  |  |  |  |  |
| **UPDRS III** | -0.074 | -0.014 |  |  |  |  |  |  |
| **STN-DBS** | -0.309 | -0.051 | -0.021 |  |  |  |  |  |
| **LEDD** | -0.206 | -0.067 | -0.383 | 0.736 |  |  |  |  |
| **ICD** | -0.030 | 0.084 | -0.066 | -0.226 | -0.415 |  |  |  |
| **Symbol search** | 0.112 | -0.088 | 0.008 | 0.095 | 0.057 | 0.084 |  |  |
| **TMT A** | -0.110 | 0.059 | 0.378 | 0.141 | 0.028 | -0.244 | -0.111 |  |
| **Stroop inhib time** | -0.186 | 0.122 | 0.262 | 0.127 | -0.201 | 0.104 | -0.443 | 0.030 |

Correlation of fixed effects for mixed effects model with reaction time as outcome; fixed effects variables listed in table 2 of supplementary material; subject ID as random effect.

**Table 4 Supplementary Material.** Average stimulation parameters for STN-DBS.

|  | overall | without ICD | with ICD |  |
| --- | --- | --- | --- | --- |
| n | 43 | 22 | 21 |  |
| Level left | 2.7 (±0.6) | 2.8 (±0.6) | 2.7 (±0.6) |  |
| Level right | 2.8 (±0.7) | 2.8 (±0.7) | 2.8 (±0.8) |  |
| Amplitude left | 2.9 (±0.8) | 2.9 (±0.9) | 2.8 (±0.7) |  |
| Amplitude right | 2.7 (±0.7) | 2.6 (±0.7) | 2.7 (±0.8) |  |
| Pulse width left | 60 (±0.0) | 60 (±0.0) | 60 (±0.0) |  |
| Pulse width right | 60 (±0.0) | 60 (±0.0) | 60 (±0.0) |  |
| Rate left | 128.9 (±2.7) | 129.0 (±3.2) | 128.8 (±2.2) |  |
| Rate right | 128.8 (±2.8) | 129 (±3.2) | 128.6 (±2.3) |  |
| Stimulation parameters for subthalamic nucleus listed for left and right STN separately. Values are means (±STD) | | | | |
